# Haploidentical stem cell transplantation with post-transplant cyclophosphamide challenges and outcome from a tertiary care center in Lebanon

**DOI:** 10.3389/frtra.2023.1149393

**Published:** 2023-06-12

**Authors:** Jean El Cheikh, Ghassan Bidaoui, Layal Sharrouf, Ammar Zahreddine, Radwan Massoud, Rita Nehme, Nabila Kreidieh, Nour Moukalled, Iman Abou Dalle, Rami Mahfouz, Ali Bazarbachi

**Affiliations:** ^1^ Division of Hematology/Oncology, Department of Internal Medicine, American University of Beirut Medical Center, Beirut, Lebanon; ^2^ Bone Marrow Transplantation Program, Department of Internal Medicine, American University of Beirut Medical Center, Beirut, Lebanon; ^3^ Department of Pathology and Laboratory Medicine, American University of Beirut, Beirut, Lebanon

**Keywords:** haploidentical hematopoietic stem cell transplantation, low- and lower-middle-income countries, hematologic malignancies, post transplant cyclophosphamide, tertiaiy care

## Abstract

This letter describes the experience of the American University of Beirut Medical Center in Lebanon with haploidentical stem cell transplant (haplo-SCT) for hematological malignancies in adult patients. Haplo-SCT made it possible through universal and rapid donor availability for most of the adult patients with leukemia or lymphoma not only in the Middle East but also globally. Moreover, the use of post-transplant cyclophosphamide (PTCy) and reduced intensity conditioning (RIC) regimens when indicated improved the outcome and decreased the toxicity of haploidentical stem cell transplant.RIC regimens also allowed its use in the elderly population. Patients from throughout the Middle East come to our center, the American university of Beirut Medical Center, to receive this transformative type of stem cell transplant. In this paper, we discuss the results of haplo-SCT with PTCy done on adult patients with hematological malignancies in our center from 2015 to 2021. The results are encouraging and show that haplo-SCT should be considered more often in the Middle Eastern countries. The subgroup analysis showed the importance of achieving complete remission of the disease prior to transplant to improve outcomes in our center. There is a paucity of literature on the outcomes of haplo-SCT in the Middle East which may contribute to the limited number of centers that offer this type of SCT. Herein, we aim to fill this gap in the hopes of encouraging the implementation of this potentially curative modality of treatment to a larger extent in the Middle East.

## Background and Introduction

Haploidentical stem cell transplant (Haplo) has made it possible that almost every allotransplant candidate has a donor (1). The outcome of Haplo has improved dramatically by using T-cell replete grafts with administration of post-transplantation cyclophosphamide (PTCy). The results of such transplants have been comparable to other historical transplant modalities (1). Locally, it has been offered at our center, the American University of Beirut Medical Center (AUBMC) in Beirut, Lebanon, to patients from throughout the Middle East and the Gulf area since 2013 (2, 3).

Autologous SCT activity in Lebanon has remained mostly stable since 2012 compared to allogeneic activity, which increased drastically (2). This increase in allogeneic stem cell transplantation (allo-SCT) can be mainly attributed to increased availability. Allo-SCT in Lebanon is limited by many factors related to the cost, third party coverage, referral and availability of donors (2). Allo-SCT with matched-related donor (MRD) is considered the best source for allo-SCT among patients with hematologic malignancies but is available for only 25% of patients (1). For the rest of the patients, matched unrelated donor (MUD) transplant is an alternative option worldwide (1). The odds of finding a MUD in international registries can range from 20% to 80% depending on patient’s ethnic background (1). However, in Lebanon and the Middle East area, the probability is less than 5% (2, 3). The low representation of the Middle East population in international registries and the lack of a public registry in the region make it impractical for almost 70% of patients with leukemia and lymphoma (2, 3). For all these reasons, only six patients received allo-SCT from MUD during those last years at our center (2, 3).

In contrast to MUD, almost 95% of the patients have at least one haploidentical donor with an average of 2.7 donors per patient (2). This universal availability of a haploidentical donor, the lower cost, and the short time needed to find the appropriate donor makes the possibility of a Haplo attainable for most patients in Lebanon (2, 3). Moreover, the use of PTCy, anti-thymocyte globulin, graft manipulation (T-cell repletion), and reduced intensity or toxicity conditioning regimen (RIC/RTC) decreased the rate of non-relapse mortality (NRM) associated with Haplo (4–10).

Haplo at AUBMC is predominantly performed for adult patients with hematological malignancies such as acute leukemia and lymphoproliferative disorders, namely Hodgkin’s and Non-Hodgkin’s lymphoma (2, 3). Haplo use in Lebanon started in 2013 (5% of allo-SCT) and reached around 40 to 50% of the allogeneic transplant activity since 2016 (2, 3). Until now, 130 (adults and pediatric) patients received Haplo at AUBMC with 117 of them from 2015 to this date (2, 3).

### Haploidentical stem cell transplant in adult patients at AUBMC from 2015 to 2021

In this letter, we describe the outcomes of Haplo with post-transplant cyclophosphamide in 99 consecutive adult patients with leukemia and lymphoma at AUBMC from January 2015 to December 2021. On average, 17 adult patients received Haplo every year. The age of the patients ranged from 17 to 79 years with a median of 41 years. One patient included in our cohort was 17-year-old at the time of transplant. The patient was referred to our center to be treated as an adult patient for the following reasons: (1) the patient’s disease and characteristics (2) the patient was close to turning 18 at the time of the transplant. The rest of our cohort was at 18-year-old at the time of the transplant. Hence, this paper is reporting the experience of adult patients. As for patients older than 60, Haplo was made possible by using RTC/RIC and updated supportive care. This procedure was performed mainly for acute leukemia, predominantly myeloid (64%), and lymphoma (24%). Almost all (98%) of these transplants were performed using peripheral blood stem cell sources (PBSC) and 63% of patients were in complete remission (CR) at the time of transplantation. Different conditioning regimens were used depending on disease type, disease status, and patient characteristics. The conditioning regimens include Thiotepa-busulfan-fludarabine with anti-thymocyte globulin (TBF- ATG) (11–13) (57%) or fludarabine cyclophosphamide with total body irradiation 2 to 4 Gy (TBI) (Flu + Cyclo + TBI) (14) (14%). Patients with relapsed/refractory (R/R) malignancies received a sequential regimen consisting of Thiotepa-etoposide-cyclophosphamide (TEC) and RIC (15) in (17%) if younger than 65 and fit for the regimen. Patients with R/R malignancies but older than 65-year-old or unfit for intensive regimens received flu-ATG-TBI (12, 13, 16) (2%), or either Clofarabine (Clo) or flu with 4 to 8 Gy total body irradiation (TBI) (17) (9%) if older than 65 years or deemed unfit for intensive regimens. All of our patients received cyclophosphamide 50 mg/kg/day at day 3 and day 5 post-transplant (18). The patients and transplant characteristics are listed in Table 1.

**Table 1:** Patient and transplant characteristics.

Characteristics	Total number of patients (*N* = 99)
Patient’s Age (years) Median (range)	41 (17–79)
Donor Age (years) Median (range)	34 (15–72)
Patient’s sex *n*(%)	
Male	61 (62)
Female	38 (38)
Donor’s sex (%)	
Male	75 (76)
Female	24 (24)
Donor-recipient sex (%)	
Male to Male	48 (48)
Female to Female	11 (11)
Male to Female	27 (27)
Female to Male	13 (13)
Donor Recipient Sex Mismatch	40 (40)
Disease type *n*(%)	
Acute Leukemia	63 (64)
Lymphoma	24 (24)
Myelodysplastic syndrome	7 (7)
Aplastic Anemia	2 (2)
Others	3 (3)
Disease status at transplant *n*(%)	
CR	65 (65)
PR	12 (12)
PD	21 (21)
Unknown	1 (1)
Conditioning regimens *n*(%)	
-TBF-ATG	57 (57)
- Flu- Cyclo-TBI	14 (14)
-TEC-RIC	17 (17)
-Clo/Flu-TBI	9 (9)
-flu- ATG—TBI	2 (2)
Stem cell source *n*(%)	
PBSC	97 (98)
BM	2 (2)
Stem cells infused median (range)	
CD34 + x 10^6^ /kg	7.39 (2.19–12.6)
CD3 + x 10^8^/kg	2.56 (0.27–10.5)
ANC engraftment day	
Median (range)	14 (11–30)
Platelet engraftment day	
Median (range)	18.5 (10–220)

MPAL, mixed phenotype acute leukemia; TBF-ATG, Thiotepa-busulfan-fludarabine-anti thymocyte globulin; Cyclo-Flu-TBI, cyclophosphamide - fludarabine -total body irradiation; Clo/Flu-TBI, Clofarabine/fludarabine - total body irradiation; Fludarabine- ATG -TBI; PBSC, peripheral blood stem cell; BM, bone marrow; GvHD, graft versus host disease; CR, complete remission; PR, partial remission; PD, progressive disease.

## Statistics and methods

This retrospective study was approved by the IRB ethics committee in our center, AUBMC. Patients who underwent haplo with post-transplant cyclophosphamide between 01, January, 2015 and 31, December, 2021 in our center were considered for this study. We collected all data retrospectively from our center’s database and used standard descriptive statistical methods to summarize it. Data collected were related to patient characteristics, such as gender and age, transplant and donor characteristics disease characteristics, such as stem cell source, disease status at transplant and conditioning regimens, and clinical events such as treatment-related complications and survival data at last follow-up. Categorical variables were compared using the X2 test, while continuous variables were compared using Student’s t-test. Progression-free survival (PFS) was defined as survival without relapse or progression of hematological disease. For patients without disease or progression, we censored their data at the last follow-up. Overall survival (OS) and NRM were defined as death from any cause and without evidence of relapse, respectively. We utilized the Kaplan-Meier method to calculate the probabilities of graft-vs.-host disease (GVHD), PFS, and OS. The cumulative incidence functions were employed to estimate the relapse incidence (RI) and NRM in a competing risk setting. A *p*-value <0.05 was considered to indicate a significant difference. All analyses were conducted using SPSS version 26.0 and R-Studio version 1.2.5019.

## Results and discussion

### GVHD and NRM

After a median follow-up of 710 days, nearly 2 years, the incidence of acute and chronic GvHD was found to be low compared to that reported in the literature in regard to Haplo with post-transplant cyclophosphamide (19, 20). Thirty four patients (34%) developed acute GvHD at the time of the last follow-up and 9 patients (9%) developed chronic GvHD at the time of the last follow-up. Seventeen (17%) of our patients developed grade III-IV acute GvHD while 9% developed grade II acute GvHD. As for the patients who developed chronic GvHD, 5 patients (5%) developed limited chronic GvHD while 4 patients (4%) developed extensive chronic GvHD. The relatively low incidence of GvHD reported here could be related to the small sample size or to the systematic concomitant use of PTCy and ATG. Infectious complications that happened in our cohort include hemorrhagic cystitis (28%), CMV reactivation (56%), clostridium difficile (17%), EBV reactivation (16%), human herpes virus 6 (7%), and central line-associated bloodstream infection (3%). As for engraftment, platelet and white blood cell engraftment failure happened in 17% and 3% of patients, respectively. NRM in our sample was 9% on day 100 after transplantation, 21% at 1 year after transplantation, and 24% at 2 years after transplantation (Table 2).

**Table 2:** Patient and transplant outcomes.

Outcome	
Disease state at last follow-up *n* (%)	
CR	57 (58%)
PR	8 (8%)
PD	34 (34%)
Acute GVHD *n* (%)	34 (34%)
II-IV	26 (26%)
II	9 (9%)
III-IV	17 (17%)
Unknown	0 (0%)
Chronic GVHD *n* (%)	9 (9%)
Limited	5 (5%)
Extensive	4 (4%)
Relapse Incidence *n* (%)	
1-year post-transplant	24%
2-year post-transplant	30%
NRM (%)	
1-year post transplantation	21%
2-year post-transplantation	24%
Disease-related mortality (%)	31%
Median OS (months)	21 months
Median PFS (months)	17 months
Overall survival at 1-year	
All patients	57%
Patients in CR at transplant	68%
Patients not in CR at transplant	39%
Overall survival at 2-year	
All patients	47%
Patients in CR at transplant	60%
Patients not in CR at transplant	24%
Progression-free survival at 1-year	
All patients	53%
Patients in CR at transplant	64%
Patients not in CR at transplant	33%
Progression-free survival at 2-year	
All patients	44%
Patients in CR at transplant	57%
Patients not in CR at transplant	21%

CR, complete remission; PR, partial response; PD, progressive disease; GVHD, graft versus host disease; NRM, non-relapse mortality; OS, overall survival; PFS, progression free survival.

### Relapse

As for relapse, the RI was 24% and 30% at 1-year and 2-years post-transplant respectively. This cumulative incidence of relapse is comparable to the cumulative incidence of relapse published by EBMT: 44% in ALL patients and 32% in AML patients after three years of transplantation (19, 20). Relapse or refractory disease related mortality turned out to be the main mortality cause in our cohort and accounted for 31% of deaths at the median follow-up time of 2-years post-transplant.

### OS and PFS

**Figure 1:**
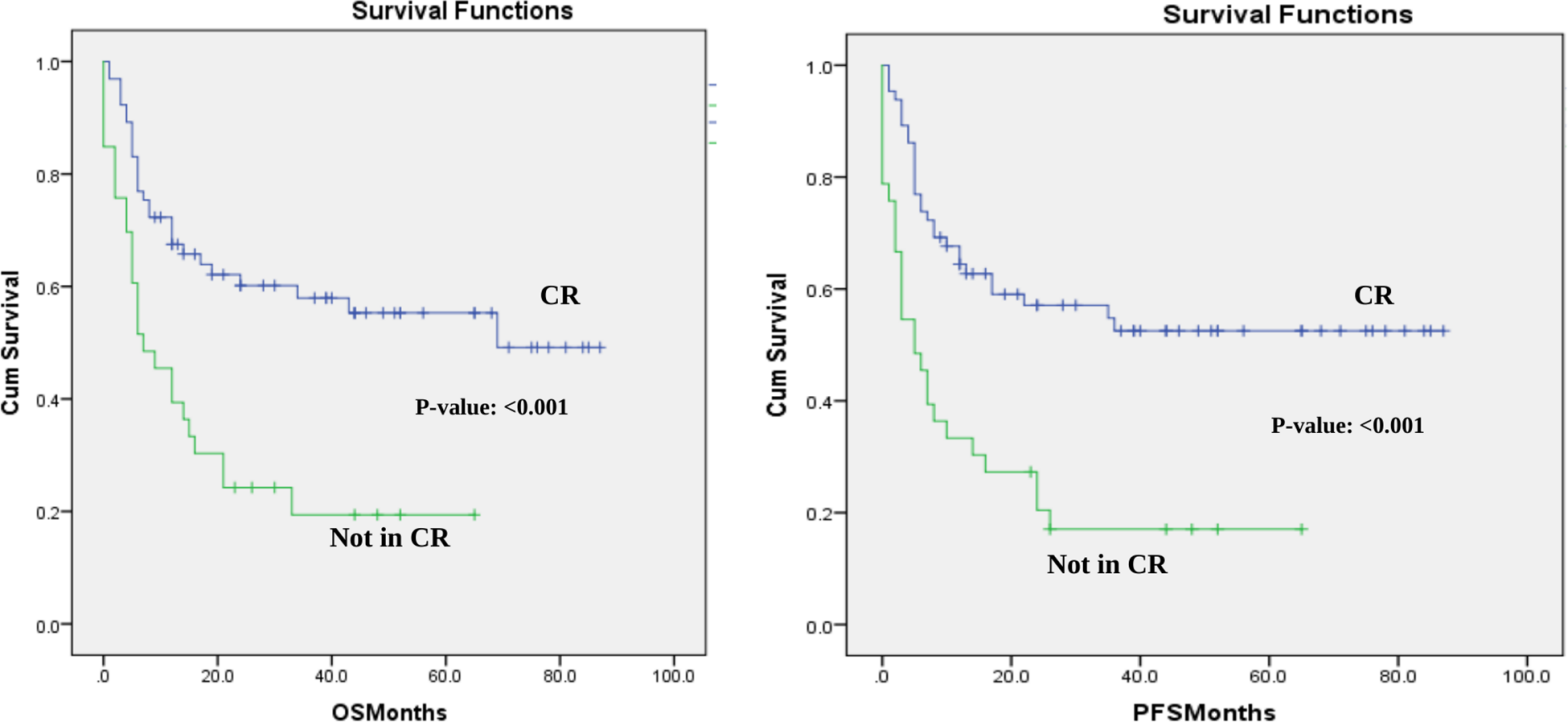
Overall survival and progression-free survival in the cohort-CR vs. not in CR at the time of transplant. OS and PFS for the whole cohort based in disease status at transplant- CR vs. not in CR at the time of transplant.

PFS was 53% and 44% at 1 year and 2 years after transplantation with a median PFS 17 months. The 1-year and 2-year posttransplant probability of OS was 57% and 47%, respectively, with median OS of 21 months. Both OS and PFS curves reached a plateau around two years after transplantation (Figure 1). These results are satisfactory since 33% of the patients in our sample had active disease at the time of the transplant, which is associated with worse outcomes (21).

## Transplant outcomes according to disease status at transplant

The analysis for the whole cohort showed that complete remission (CR) at transplant significantly affected the median OS, median PFS, and NRM in our cohort. The median OS for patients who had CR at the time of transplantation not reached compared to only 7 months in patients who did not have CR at transplant.(*p*-value: <0.001) (Figure 1). The median PFS in the CR subgroup was not reached while in the other group it was only 5 months (*p*-value: <0.001) (Figure 1). Finally, patients who were in CR had a 4% less NRM rate compared to the other group (17% vs. 21%; *p*-value: 0.002). These results show that the survival outcomes in the whole cohort can be partly explained with the worse prognosis in patients who had the transplant with residual or active disease.

**Figure 2:**
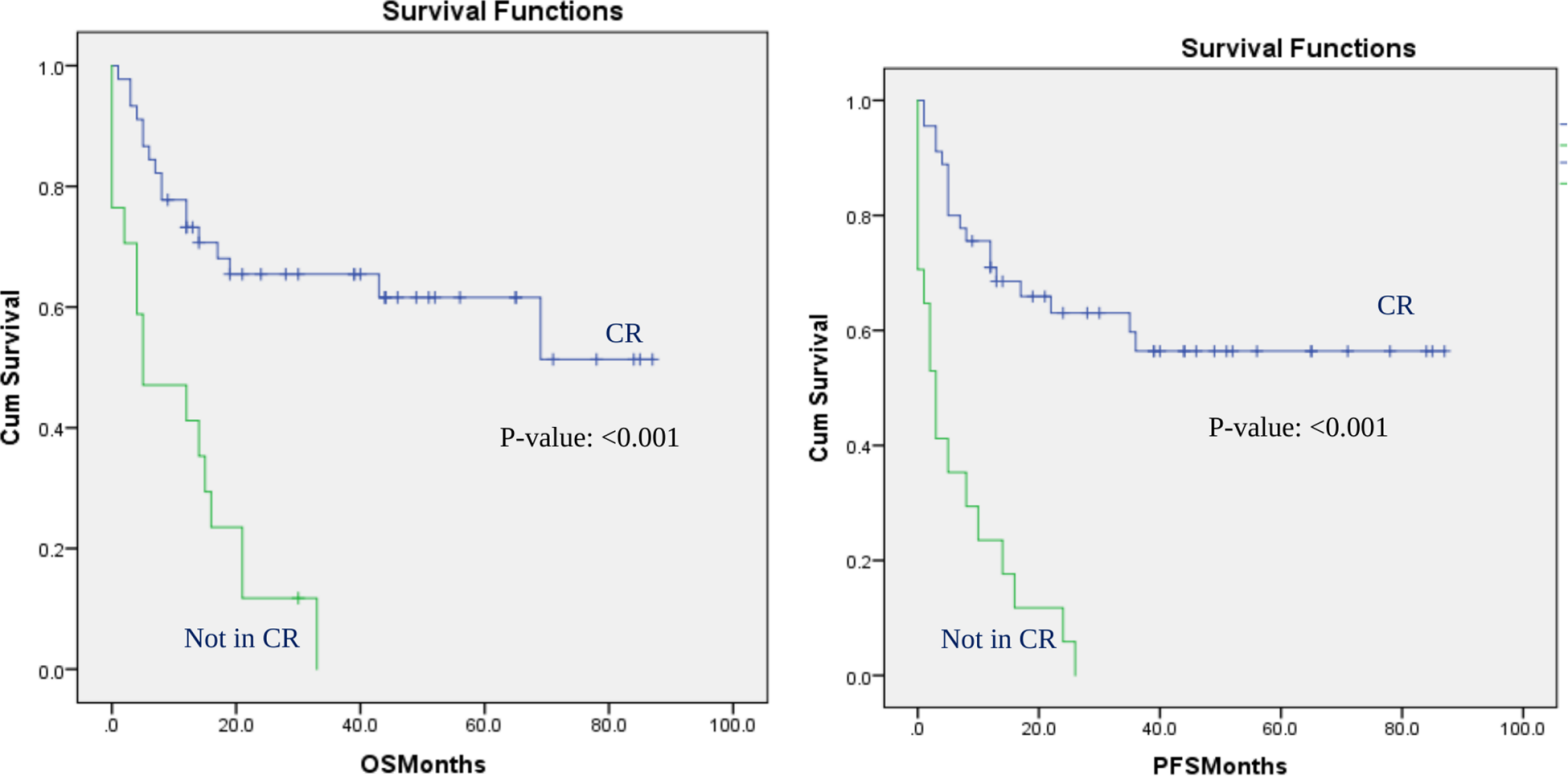
Overall survival and progression-free survival in patients with AML: CR vs. not in CR at the time of transplant. OS and PFS for AML patients based on disease status at transplant: CR vs. not in CR at the time of transplant.

This effect was still significant for all outcomes when the analysis was performed in patients with AML. In patients with AML, patients who were in complete remission at the time of transplant did not even reach the median OS or median PFS. Patients with AML who were not in CR had only a median OS of 5 months (*P*-value: <0.001) and a median PFS of 3 months (*p*-value: <0.001) (Figure 2). Patients with AML who had the transplant at CR had 13% less NRM rate than patients who had the transplant with residual disease (16% vs. 29% *p*-value: <0.001). On the other hand, the subgroup analysis did not show a significant effect of disease status at transplantation on any of the outcomes in the cohort of patients with lymphoma, although a positive trend was observed. In our center, an allogeneic transplant is administered for patients with lymphoma only after other therapy has failed, which could have affected the results of the lymphoma subgroup analysis.

Finally, a subgroup analysis was performed for patients who received TBF, the most utilized myeloablative regimen in our cohort. CR at the time of transplantation was significantly associated with a higher median PFS in this subgroup (36 months vs. 10 months; *p*-value: 0.044). The median OS also showed a positive trend but did not reach significance.

## Conclusion

In conclusion, Haplo has been and is still used successfully at AUBMC to fill the gap in adult patients with acute leukemia and lymphoma, owing to the universal and rapid donor availability, cost, and outcomes. Our analysis showed that CR should be achieved before transplant to improve transplantation in the center. Since almost all of our patients have at least one haploidentical donor, it should now be possible to and offer this potentially curative option for patients with high-risk hematologic disease (1).

## Data Availability

The original contributions presented in the study are included in the article, further inquiries can be directed to the corresponding author/s.

## References

[1] Khan MA Bashir Q Chaudhry QUN Ahmed P Satti TM Mahmood SK. Review of haploidentical hematopoietic cell transplantation. J Glob Oncol. (2018) 4:1–13. 10.1200/jgo.18.00130 PMC7010419 30521413

[2] Ali B Ammar Z Radwan M Jean EC Colette H Fadi N Trends in hematopoietic stem cell transplant activity in Lebanon. Trends Hematopoietic Stem Cell Transplant Activ Lebanon. (2017) 10:315–20. 10.1016/j.hemonc.2017.05.003 28641098

[3] Bazarbachi A Hatoum H Mugharbel A Otrock Z Yassine N Muwakkit S Hematopoietic stem cell transplantation in Lebanon: first comprehensive report. Bone Marrow Transplant. (2008) 42(Suppl 1):S96–102. 10.1038/bmt.2008.128 18724316

[4] Im A Rashidi A Wang T Hemmer M MacMillan ML Pidala J Risk factors for graft-versus-host disease in haploidentical hematopoietic cell transplantation using post-transplant cyclophosphamide. Biol Blood Marrow Transplant. (2020) 26(8):1459–68. 10.1016/j.bbmt.2020.05.001 32434056 PMC7391266

[5] Bashey A Zhang X Sizemore CA Manion K Brown S Holland HK T-cell-replete HLA-haploidentical hematopoietic transplantation for hematologic malignancies using post-transplantation cyclophosphamide results in outcomes equivalent to those of contemporaneous HLA-matched related and unrelated donor transplantation. J Clin Oncol. (2013) 31(10):1310–6. 10.1200/JCO.2012.44.3523 23423745

[6] Ciurea SO Zhang MJ Bacigalupo AA Bashey A Appelbaum FR Aljitawi OS Haploidentical transplant with posttransplant cyclophosphamide vs matched unrelated donor transplant for acute myeloid leukemia. Blood. (2015) 126(8):1033–40. 10.1182/blood-2015-04-639831 26130705 PMC4543223

[7] Kanate AS Mussetti A Kharfan-Dabaja MA Ahn KW DiGilio A Beitinjaneh A Reduced-intensity transplantation for lymphomas using haploidentical related donors vs. HLA-matched unrelated donors. Blood. (2016) 127(7):938–47. 10.1182/blood-2015-09-671834 26670632 PMC4760094

[8] Kasamon YL Bolaños-Meade J Prince GT Tsai HL McCurdy SR Kanakry JA Outcomes of nonmyeloablative HLA-haploidentical blood or marrow transplantation with high-dose post-transplantation cyclophosphamide in older adults. J Clin Oncol. (2015) 33(28):3152–61. 10.1200/JCO.2014.60.4777 26261255 PMC4582145

[9] Wang Y Wu DP Liu QF Xu LP Liu KY Zhang XH Low-dose post-transplant cyclophosphamide and anti-thymocyte globulin as an effective strategy for GVHD prevention in haploidentical patients. J Hematol Oncol. (2019) 12(1):88. 10.1186/s13045-019-0781-y 31481121 PMC6724335

[10] Aversa F Tabilio A Velardi A Cunningham I Terenzi A Falzetti F Treatment of high-risk acute leukemia with T-cell-depleted stem cells from related donors with one fully mismatched HLA haplotype. N Engl J Med. (1998) 339(17):1186–93. 10.1056/NEJM199810223391702 9780338

[11] Duléry R Bastos J Paviglianiti A Malard F Brissot E Battipaglia G Thiotepa, busulfan, and fludarabine conditioning regimen in T cell-replete HLA-haploidentical hematopoietic stem cell transplantation. Biol Blood Marrow Transplant. (2019) 25(7):1407–15. 10.1016/j.bbmt.2019.02.025 30871978

[12] Salas MQ Atenafu EG Law AD Lam W Pasic I Chen C Lower dose of ATG combined with post-transplant cyclophosphamide for HLA matched RIC alloHCT is associated with effective control of GVHD and less viral infections. Leuk Lymphoma. (2021) 62(14):3373–83. 10.1080/10428194.2021.1966781 34435547

[13] Xu X Yang J Cai Y Li S Niu J Zhou K Low dose anti-thymocyte globulin with low dose posttransplant cyclophosphamide (low dose ATG/PTCy) can reduce the risk of graft-versus-host disease as compared with standard-dose anti-thymocyte globulin in haploidentical peripheral hematopoietic stem cell transplantation combined with unrelated cord blood. Bone Marrow Transplant. (2021) 56(3):705–8. 10.1038/s41409-020-01047-2 32873913 PMC7943423

[14] Luznik L O’Donnell PV Symons HJ Chen AR Leffell MS Zahurak M HLA-haploidentical bone marrow transplantation for hematologic malignancies using nonmyeloablative conditioning and high-dose, posttransplantation cyclophosphamide. Biol Blood Marrow Transplant. (2008) 14(6):641–50. 10.1016/j.bbmt.2008.03.005 18489989 PMC2633246

[15] Duléry R Ménard AL Chantepie S El-Cheikh J François S Delage J Sequential conditioning with thiotepa in T cell- replete hematopoietic stem cell transplantation for the treatment of refractory hematologic malignancies: comparison with matched related, haplo-mismatched, and unrelated donors. Biol Blood Marrow Transplant. (2018) 24(5):1013–21. 10.1016/j.bbmt.2018.01.005 29337223

[16] Cao J Pei R Lu Y Zheng Z Yuan Z Li D Fludarabine and antithymocyte globulin-based conditioning regimen combined with post-transplantation cyclophosphamide for haploidentical allogeneic hematopoietic stem cell transplantation in patients with high-risk acute myeloid leukemia and myelodysplastic syndrome. Curr Res Transl Med. (2023) 71(1):103360. 10.1016/j.retram.2022.103360 36427418

[17] Cheikh J E Bidaoui G Atoui A Terro K Sharrouf L Zahreddine A Clofarabine and total body irradiation (TBI) as conditioning regimen for allogeneic stem cell transplantation in high-risk acute leukemia patients: a two-center retrospective cohort study. Bone Marrow Transplant. (2023). 10.1038/s41409-023-01947-z 36914730

[18] Ruggeri A Labopin M Battipaglia G Chiusolo P Tischer J Diez-Martin JL Timing of post-transplantation cyclophosphamide administration in haploidentical transplantation: a comparative study on behalf of the acute leukemia working party of the European society for blood and marrow transplantation. Biol Blood Marrow Transplant. (2020) 26(10):1915–22. 10.1016/j.bbmt.2020.06.026 32645444

[19] Piemontese S Boumendil A Labopin M Schmid C Ciceri F Arcese W Leukemia relapse following unmanipulated haploidentical transplantation: a risk factor analysis on behalf of the ALWP of the EBMT. J Hematol Oncol. (2019) 12(1):68. 10.1186/s13045-019-0751-4 31272508 PMC6610936

[20] Adhikari J Gyawali B Sharma P Bhatt VR. Outcomes of haploidentical transplant compared with matched donor allogeneic stem cell transplant. Future Oncol. (2017) 13(10):935–44. 10.2217/fon-2016-0443 27935324

[21] Ahmed SO El Fakih R Elhaddad A Hamidieh AA Altbakhi A Chaudhry QUN Strategic priorities for hematopoietic stem cell transplantation in the EMRO region. Hematol Oncol Stem Cell Ther. (2021) 16(3):162–9. 10.1016/j.hemonc.2021.09.006 34688625

